# Cellular alterations identified in pluripotent stem cell-derived midbrain spheroids generated from a female patient with progressive external ophthalmoplegia and parkinsonism who carries a novel variation (p.Q811R) in the POLG1 gene

**DOI:** 10.1186/s40478-019-0863-7

**Published:** 2019-12-16

**Authors:** Margarita Chumarina, Kaspar Russ, Carla Azevedo, Andreas Heuer, Maria Pihl, Anna Collin, Eleonor Åsander Frostner, Eskil Elmer, Poul Hyttel, Graziella Cappelletti, Michela Zini, Stefano Goldwurm, Laurent Roybon

**Affiliations:** 1 Cell Stem Cell Laboratory for CNS Disease Modeling, Department of Experimental Medical Science, Lund University, 22184 Lund, Sweden; 2 MultiPark and the Lund Stem Cell Center, Lund University, 22184 Lund, Sweden; 3 Behavioural Neuroscience Laboratory, Department of Experimental Medical Science, Lund University, 22184 Lund, Sweden; 4 Department of Veterinary and Animal Sciences, Faculty of Health and Medical Sciences, University of Copenhagen, 1870 Copenhagen, Denmark; 5 Office for Medical Services/Division of Laboratory Medicine, Department of Clinical Genetics and Pathology, Lund, Sweden; 6 Department of Clinical Sciences Lund, Mitochondrial Medicine, Lund University, 22184 Lund, Sweden; 7 Department of Biosciences, Università degli Studi di Milano, via Celoria 26, I-20133 Milan, Italy; 8 Center of Excellence on Neurodegenerative Diseases, Università degli Studi di Milano, via Balzaretti, I-20133 Milan, Italy; 9 Parkinson Institute, ASST Pini-CTO, via Bignami 1, 20126 Milan, Italy

**Keywords:** Parkinson’s disease, POLG1, iPSCs, Midbrain spheroids, Proteomics, MAO-B, Glycolysis, Alpha-synuclein

## Abstract

Variations in the POLG1 gene encoding the catalytic subunit of the mitochondrial DNA polymerase gamma, have recently been associated with Parkinson’s disease (PD), especially in patients diagnosed with progressive external ophthalmoplegia (PEO). However, the majority of the studies reporting this association mainly focused on the genetic identification of the variation in *POLG1* in PD patient primary cells, and determination of mitochondrial DNA copy number, providing little information about the cellular alterations existing in patient brain cells, in particular dopaminergic neurons. Therefore, through the use of induced pluripotent stem cells (iPSCs), we assessed cellular alterations in novel p.Q811R *POLG1* (POLG1^Q811R^) variant midbrain dopaminergic neuron-containing spheroids (MDNS) from a female patient who developed early-onset PD, and compared them to cultures derived from a healthy control of the same gender. Both *POLG1* variant and control MDNS contained functional midbrain regionalized TH/FOXA2-positive dopaminergic neurons, capable of releasing dopamine. Western blot analysis identified the presence of high molecular weight oligomeric alpha-synuclein in POLG1^Q811R^ MDNS compared to control cultures. In order to assess POLG1^Q811R^-related cellular alterations within the MDNS, we applied mass-spectrometry based quantitative proteomic analysis. In total, 6749 proteins were identified, with 61 significantly differentially expressed between POLG1^Q811R^ and control samples. Pro- and anti-inflammatory signaling and pathways involved in energy metabolism were altered. Notably, increased glycolysis in POLG1^Q811R^ MDNS was suggested by the increase in PFKM and LDHA levels and confirmed using functional analysis of glycolytic rate and oxygen consumption levels. Our results validate the use of iPSCs to assess cellular alterations in relation to PD pathogenesis, in a unique PD patient carrying a novel p.Q811R variation in *POLG1*, and identify several altered pathways that may be relevant to PD pathogenesis.

## Introduction

Parkinson’s Disease (PD) is the most prevalent neurodegenerative movement disorder associated with progressive dopaminergic neuronal loss in the *substantia nigra pars compacta* (*SNpc*). Despite extensive research efforts, the underlying cause of PD largely remains unknown [18]. However, a number of cellular processes, including mitochondrial function, have been implicated in the aetiology of PD [21, 25, 36]. First insights into the role of mitochondria in PD came from the observation that 1-methyl-4-phenyl-1,2,3,6-tetrahydropyridine (MPTP) induces dopamine neuron death through inhibition of the complex I of the mitochondrial respiratory chain, resulting in parkinsonism [51]. Later, a number of genes associated with familial forms of PD were identified to play a role in the maintenance of mitochondrial function, further suggesting its involvement in PD pathogenesis [1, 52]. Moreover, mitochondrial DNA (mtDNA) variations and depletion in PD have been extensively investigated, although without definitive conclusions [48]. Notably, higher levels of mtDNA deletions in *SNpc* of both PD and aged brains have been reported [9, 40], and reduced mtDNA copy number has been suggested as a biomarker of PD [19, 54].

The mitochondrial DNA polymerase gamma encoded by the nuclear POLG1 gene, is responsible for the synthesis of mtDNA [26]. Association of variants of *POLG1* with parkinsonism was first reported in 2004, a study of seven families with *POLG1*-related progressive external ophthalmoplegia (PEO) revealed a co-segregation of parkinsonism with *POLG1* variations, with the age of onset of parkinsonism varying between 36 and 75 years; post-mortem examination of two patients showed loss of pigmented dopaminergic neurons in the *SNpc*, but no presence of Lewy Body pathology [44]. In the same year, another study described a family with PEO, neuropathy and late-onset parkinsonism [45]. *POLG1*-related PEO can occur in either autosomal recessive (arPEO) form, characterized by ptosis and ophthalmoparesis, or autosomal dominant (adPEO) form that can include symptoms of myopathy, hearing loss, cataracts, ovarian failure, axonal neuropathy, ataxia, depression and parkinsonism [55]. Since then, additional cases of *POLG1*-associated parkinsonism have been described, often secondary to PEO and ataxia, with late age-of-onset, complete or partial L- DOPA response, and Lewy body pathology, but only in some cases [10, 56, 63]. However, early-onset *POLG1* variant-related parkinsonism has also been observed in patients without PEO [17, 47]. Interestingly, a study of eleven patients with *POLG1* variant-related encephalopathy revealed﻿ severe nigral neuronal loss and nigrostriatal depletion through DAT imaging, without any clinical signs of parkinsonism [74].

Here, we present a case study of a female patient with a novel variation in *POLG1* (p.Q811R), who was diagnosed with early-onset parkinsonism, and subsequently, adPEO. We employed a recently developed protocol to differentiate patient-derived induced pluripotent stem cells (iPSCs) into 3D midbrain dopaminergic neuron-containing spheroids (MDNS) [35] to examine POLG1^Q811R^ cellular alterations.

## Results

### Case description

The patient is a woman who developed at 24 years of age (2008) resting tremor at right arm, and afterwards resting tremor and bradykinesia to the right-side of the body. At 27 years of age, she came to the Parkinson Institute, Milano, Italy, where clinical diagnosis of Parkinson’s Disease was established, confirmed by Dopamine transporter SPECT imaging, which revealed reduction mainly in the left striatum, while brain MRI was unremarkable. She started dopaminergic therapy with excellent motor response. She also presented cataract at right eye at 17 years old, treated with surgery, and Premature Ovarian Failure at 28 years of age. In the following 10 years, symptoms of PD increased gradually including complex features of fatigue and myopathy without pathological lesions at Electromyography (EMG).

Genetic analysis of the major PD genes disclosed the presence of a heterozygous genetic variant at exon 15 of the POLG1 gene (NM_002693.2): c.2432A > G (p.Gln811Arg). This missense variant is novel, and it has never been previously described in patients with POLG1 related syndromes, and it is not present in GnomAD. The predictions based on the sequence are largely in favor of a pathogenic effect (DANN, DEOGEN2, EIGEN, FATHMM-MKL, M-CAP, MVP, MutationAssessor, MutationTaster, PrimateAI, REVEL and SIFT have a pathogenic prediction vs no benign predictions; https://varsome.com).

The clinical picture (early onset cataract, POF, early onset PD, fatigue and myopathy) and the presence of probable pathogenetic mutation on the POLG1 gene indicated a diagnosis of AUTOSOMAL DOMINANT PROGRESSIVE EXTERNAL OPHTHALMOPLEGIA (OMIM: #157640).

### Generation and characterization of iPSCs from a PD patient carrying the variation p.Q811R in *POLG1*

**Fig. 1:**
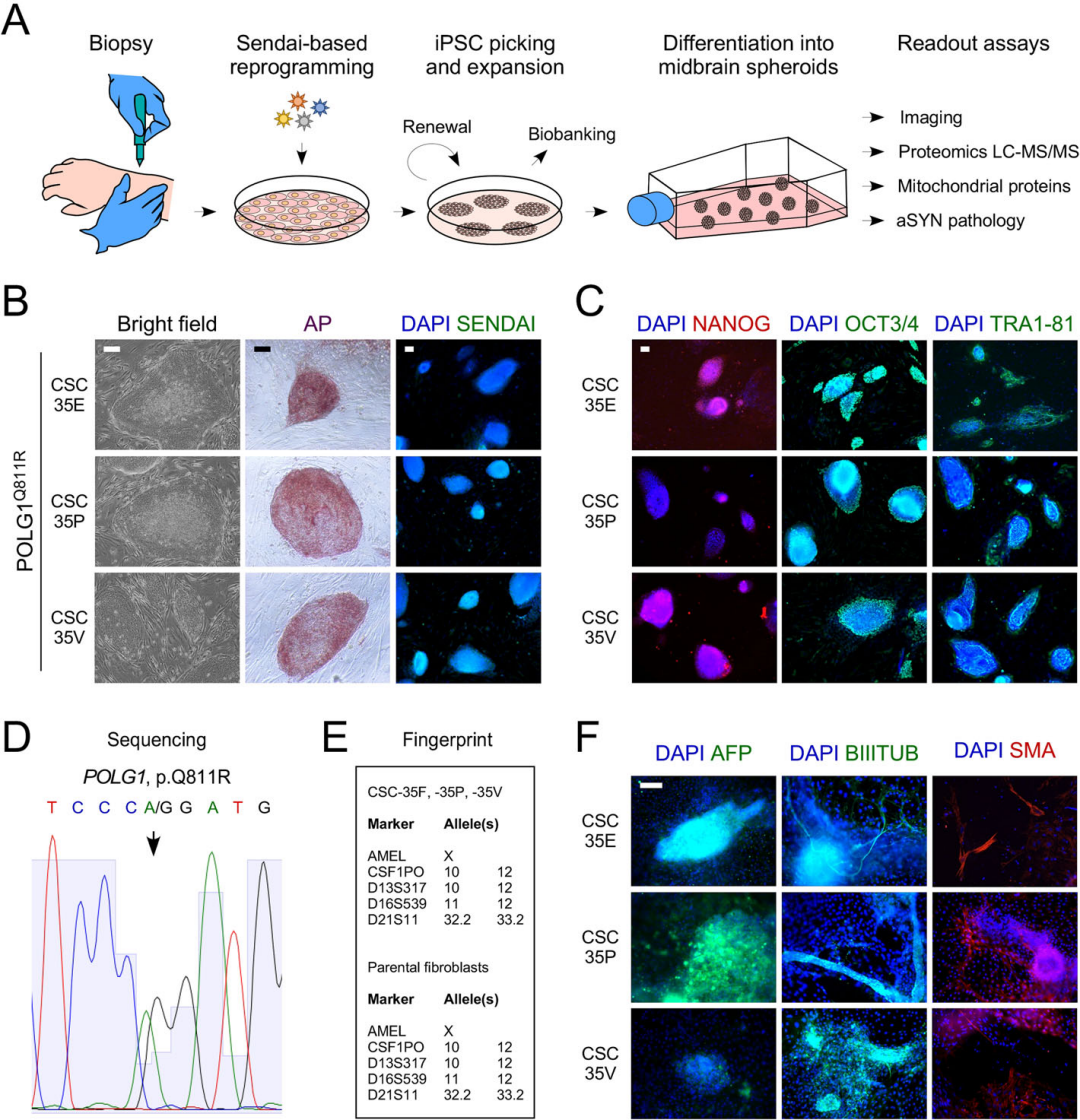
Generation and characterization of iPSCs from a PD patient carrying the variation p.Q811R in *POLG1.* **a** Schematic representation of iPSC generation and experimental workflow. **b** Bright field images showing iPSC colony formation, alkaline phosphatase activity, and loss of Sendai virus in three iPSC clones derived from the *POLG1* p.Q811R variant patient. Scale bars: 100 μm. **c** *POLG1* p.Q811R variant iPSC clones stained for the pluripotent markers NANOG, OCT4, TRA1–81. Scale bar: 100 μm. **d** DNA sequencing analysis confirming the presence of p.Q811R (c.2432 A > G). **e** DNA fingerprinting confirming genetic equivalency to parental fibroblasts. **f** Immunocytochemistry for alpha-fetoprotein (AFP), beta-III-tubulin (BIIITUB) and smooth muscle actin (SMA), confirming formation of all three germ layers. Scale bar: 100 μm

To generate iPSCs, fibroblasts were collected from the POLG1^Q811R^ patient by punch skin biopsy, grown, and reprogrammed by Sendai virus delivery of the four Yamanaka factors OCT-3/4, KLF-4, SOX-2 and c-MYC (Fig. 1a). After the expansion and bio-banking of several reprogrammed clonal iPSC cell lines, three clones, namely lines CSC-35E, CSC-35P and CSC-35 V, were selected for further expansion and characterization. For each line, quality control was carried out according to previously described methods [31]. The three iPSC lines demonstrated alkaline phosphatase activity, loss of Sendai virus with passaging, and expression of nuclear and cell surface markers OCT4, NANOG and TRA-1–81, specific to pluripotent stem cell stage (Fig. 1b, c). DNA sequencing confirmed the presence of an A > G substitution at position c.2432 resulting in the p.Q811R variation (Fig. 1c). Cells had a normal karyotype and could spontaneously differentiate into derivatives of the three germ layers, confirmed by the presence of endodermal marker alpha-fetoprotein, mesodermal marker smooth muscle actin, and ectodermal marker beta-III-tubulin (Fig. 1d, e). IPSC lines (CSC-37 N and CSC-37R) derived from a non-demented healthy individual of the same gender, were used as a control (Russ et al.; Pomeshchik et al.; manuscripts in revision).

### Generation and characterization of MDNS from POLG1^Q811R^ variant and healthy control iPSCs

**Fig. 2:**
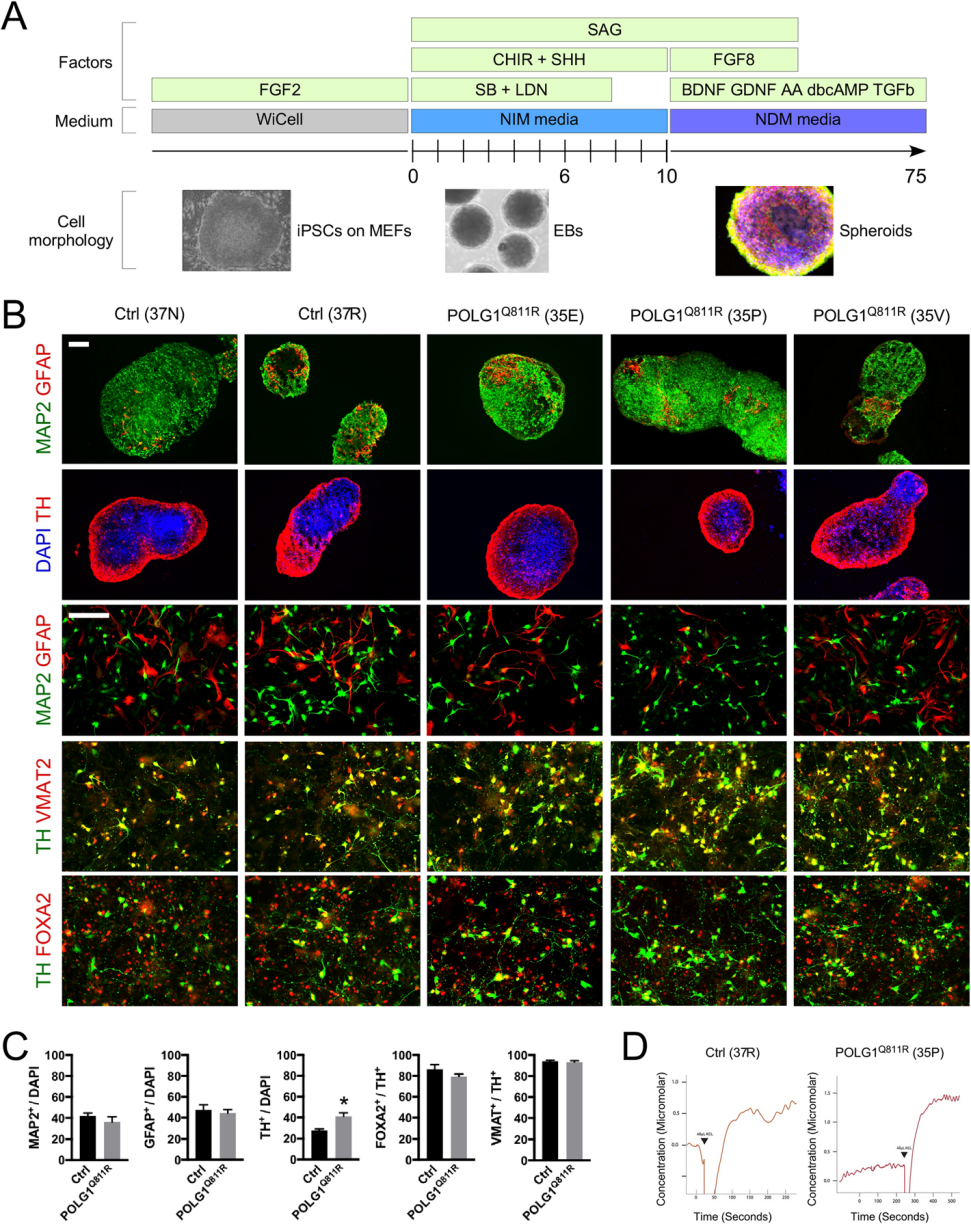
Generation and characterization of MDNS from POLG1^Q811R^ variant and healthy control iPSCs. **a** Schematic model illustrating the differentiation strategy employed to obtain MDNS. Images illustrate cell morphology at each stage. **b** Immunostaining for brain cell subtype markers GFAP and MAP2, midbrain marker FOXA2 and dopaminergic neuronal markers TH and VMAT, on cryosectioned MDNS aged 100 DIV and dissociated cultures aged 106 DIV. Nuclei are counterstained with DAPI. Scale bars: 100 μm. **c** Quantification of MAP2^+^, GFAP^+^ and TH^+^ cells relative to total number of DAPI-labeled cells, FOXA2^+^ and VMAT^+^ cells relative to TH-labeled cells in POLG1 variant and healthy control cultures. Results are presented as ± SEM; *n* = 4 independent experiments; * indicates *p* < 0.05; unpaired *t*-test. **d** Electrochemical chronoamperometric recordings showing DA release upon KCL stimulation by *POLG1* variant and healthy control MDNS dissociated cultures

To differentiate the iPSC lines into MDNS, we employed dual inhibition of SMAD signaling through the addition of SB-431542 and LDN-193189 to neuralize the cells, and CHIR-99021 and sonic hedgehog to caudalize and ventralize them, respectively (Fig. 2a [30];). Addition of FGF8 was further used to instruct a midbrain floor plate identity [59]. It has been recently reported that iPSC-derived organoids form dopaminergic neurons contained neuromelanin (NM) granules if exposed to L-DOPA or dopamine (DA) [35]. Therefore, we supplemented the cultures with 50 μM of DA at later stages of the differentiation, in combination with neurotrophic factors. After a period of 100 days in vitro (DIV), MDNS were collected for proteomic analysis, and cryo-sectioned and immune-stained (Fig. 2b). MDNS predominantly contained MAP2-positive neurons expressing tyrosine hydroxylase (TH), the rate limiting enzyme in the production of DA (Fig. 2b), and few GFAP-positive glia. To quantify the number of dopaminergic neurons, MDNS were dissociated into single cell suspension, and grown for 6 days on adherent surface (Fig. 2b, c, and Additional file 2: Figure S1). Immunocytochemistry analysis of the dissociated cultures revealed that both POLG1^Q811R^ and control cultures were composed of approximately equal proportion of GFAP+ astrocytes and MAP2+ neurons (Additional file 2: Figure S1). Around 30 to 40% of the DAPI-positive cells expressed TH, out of which 80 to 100% co-expressed the vesicular monoamine transporter 2 (VMAT2), necessary for DA transmission, as well as the midbrain-related transcription factor FOXA2. Overall, there was little variability in term of differentiation capability, between the different clones studied (Additional file 2: Figure S1). Interestingly, there was a higher number of TH-positive neurons in POLG1^Q811R^ MDNS compare to control cultures. Additionally, we performed electrochemical recordings for two of the lines studied, namely 37R (p24) and 35P (p16), to confirm the functionality of the dopaminergic neurons present in culture, through release of DA upon stimulation (Fig. 2d).

### Western blot analysis reveals presence of oligomeric species of α-synuclein of high molecular weight in POLG1^Q811R^ variant MDNS

**Fig. 3:**
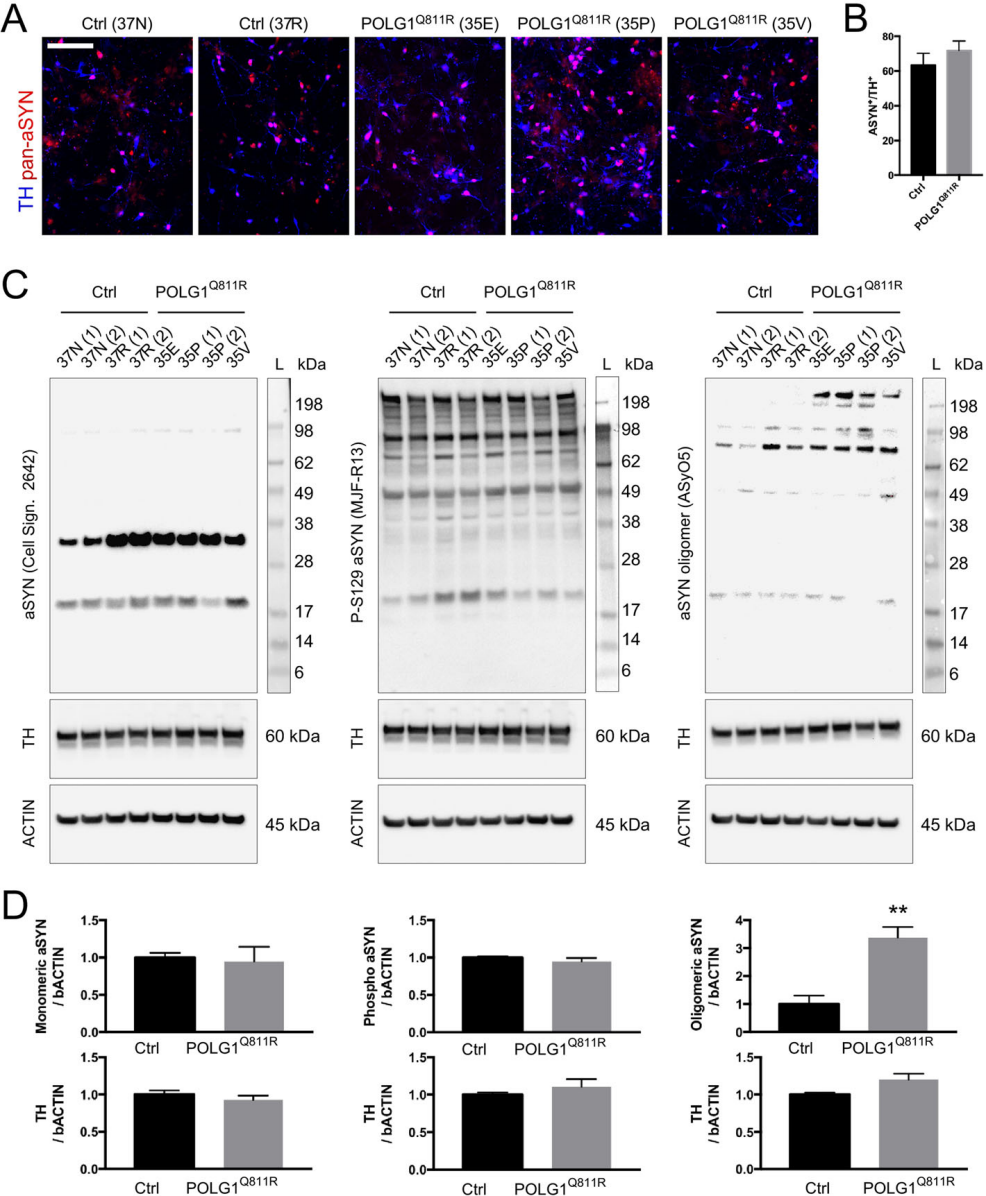
Western blot analysis reveals presence of oligomeric species of alpha-synuclein of high molecular weight in POLG^Q811R^ variant MDNS. **a** Immunostaining of pan-α-synuclein in POLG1 variant and healthy control MDNS dissociated cells 106 DIV. Scale bar: 100 μm. **b** Quantification of aSYN-positive cells relative to total number of DAPI-labeled cells, in POLG1 variant and healthy control cultures. Results are presented as ± SEM; *n* = 4 independent experiments; unpaired *t*-test. **c** Expression of monomeric alpha-synuclein, phosphorylated (pS129) alpha-synuclein, and oligomeric (ASyO5) alpha-synuclein in *POLG1* variant and healthy control MDNS. **d** Protein expression levels from (**b**) normalized to b-ACTIN. Results are presented as ± SEM; *n* = 4 independent experiments. ** indicates *p* < 0.01; unpaired *t*-test

Since PD pathogenesis is widely associated with α-synuclein (aSYN) aggregation [69], we decided to first assess the level of aSYN in the MDNS, prior to examining the changes in the proteome. Immunocytochemistry analysis of dissociated MDNS showed presence of aSYN in TH-positive dopaminergic neurons for both control and POLG1^Q811R^ cultures (Fig. 3a and b). Western blot analysis revealed no significant differences in the levels of monomeric aSYN or phosphorylated aSYN at position serine 129, between POLG1^Q811R^ and control MDNS (Fig. 3c and d). However, immunoblot analysis for oligomeric aSYN (ASyO5) revealed the presence of high molecular weight oligomers (approx. 190 kDa) in POLG1^Q811R^ MDNS, compared to control samples (Fig. 3c and d).

### Analysis of the proteome of POLG1^Q811R^ variant and healthy control MDNS reveals alterations in several pathways linked to PD pathogenesis

**Fig. 4:**
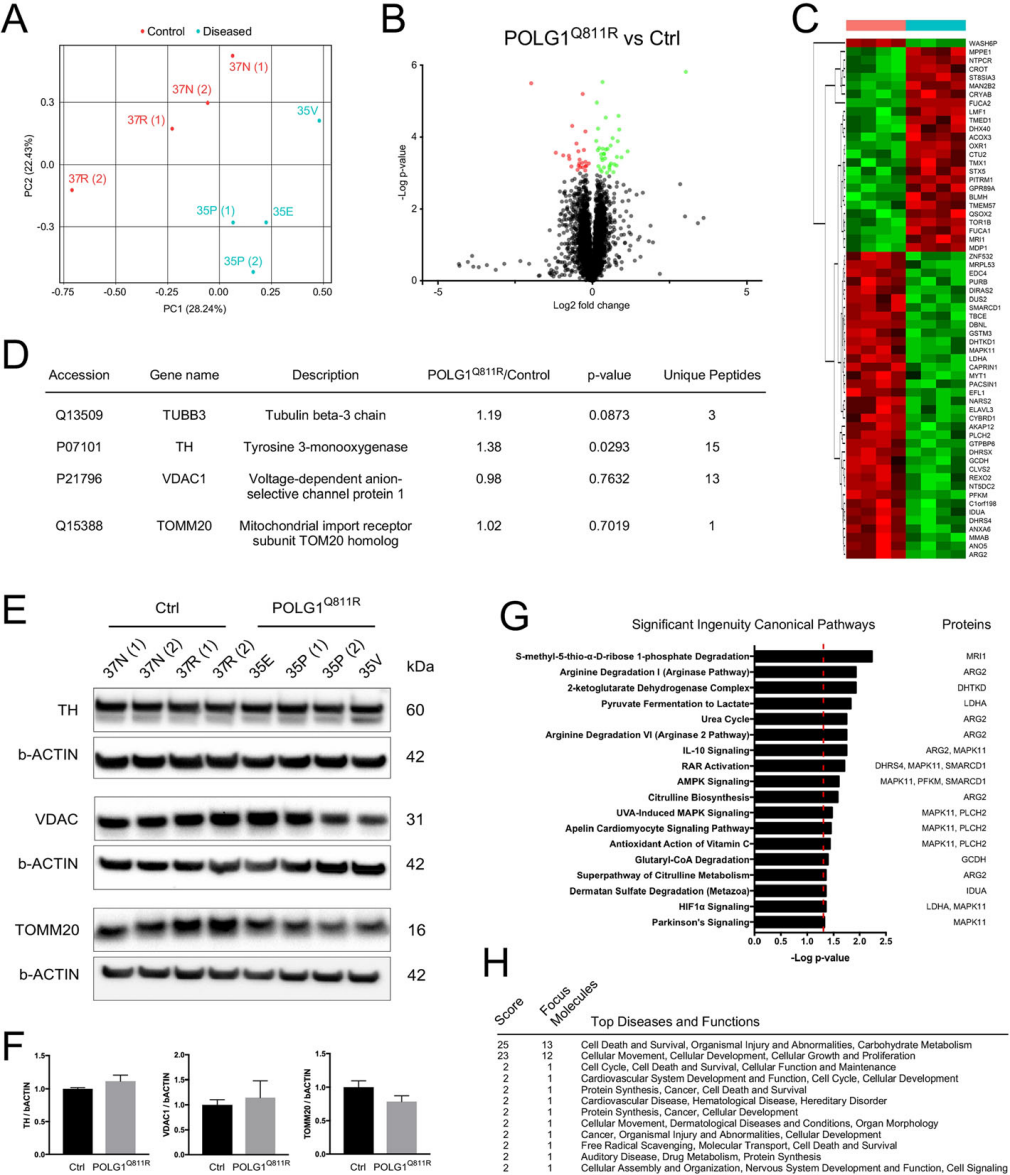
Analysis of the proteome of POLG1^Q811R^ variant and healthy control MDNS reveals alterations in several pathways linked to PD pathology. **a** Protein expression variances between POLG1 variant and healthy control MDNS as principal component analysis (PCA). The first two axes (PCA axis 1 and 2) of the PCA explain 51% of the variance. Red and turquoise dots represent healthy control and POLG1 variant clones/differentiations, respectively. **b** Volcano plot displaying differentially expressed proteins in *POLG1* variant and healthy control MDNS. Y-axis corresponds to the log10 (*p*-value), and X-axis displays the log2 (fold change) values. The cut-off for significantly altered protein levels is set at *P* < 0.001. The red dots represent significantly down-regulated proteins, and the green dots represent significantly up-regulated proteins. **c** Heat map representing color-coded expression levels (red for down-regulated and green for up-regulated) of proteins differentially expressed between *POLG1* variant (cyan) and healthy control (orange) MDNS, with *P* < 0.001. **d** Abundance levels for neuronal markers TUBB3 and TH and mitochondrial markers TOMM20 and VDAC1 in *POLG1* variant and healthy control MDNS identified by LC MS/MS quantitative proteomics. **e** and **f** Western blot confirming no difference in the levels of TH, TOMM20 and VDAC1 between *POLG1* variant and healthy control MDNS. Protein expression levels are normalized to b-ACTIN. Results are presented as ± SEM, n = 4 independent experiments. **g** Ingenuity Pathways analysis (IPA) identifying regulated pathways for differently expressed proteins POLG1 variant and healthy control MDNS. The figure shows the altered canonical pathways in IPA canonical pathways analysis. The Y-axis shows the regulated pathways and the X-axis shows the –log(p-value). A cut-off of *P* = 0.001 was used for the proteins to be included in the analyses. **h** Top diseases and functions identified based on the differentially expressed proteins, with indication of scores and focus molecules for each category

Mass-spectrometry based proteomic analysis allows for a robust characterization of disease-specific protein signatures and identification of altered molecular pathways [11]. Therefore, to gain insights into the cellular networks and pathways that could potentially be altered in POLG1^Q811R^ MDNS, we performed liquid chromatography-tandem mass spectrometry (LC-MS/MS). Quantitative proteomic profiling identified a total of 6749 proteins in POLG1^Q811R^ and control MDNS, 421 of which were mitochondrial (Additional file 1: Table S1). Overall, POLG^Q811R^ cultures showed differential protein expression profile compared to control samples, as confirmed by Principal Component Analysis (Fig. 4a). In order to decrease the number of false positive hits, we only considered protein change that had a *P* value < 0.001 as significant.

Using this threshold cut-off, we found 61 proteins significantly altered between POLG1^Q811R^ and control MDNS, with 24 down-regulated and 37 up-regulated proteins in POLG1^Q811R^ MDNS (Fig. 4b, c). The average abundance of neuronal markers TH and TUBB3 was not significantly altered between control and diseased groups, and the levels of mitochondrial marker VDAC1 and the mitochondrial import receptor subunit TOM20 did not show any changes (Fig. 4d). Western blot analysis also confirmed no difference between the levels of TH, VDAC1 and TOM20 (Fig. 4e, f), further confirming that control and POLG^Q811R^ MDNS had comparable neuronal composition and number of mitochondria, although there was a trend for increase in TH and a slightly higher number of TH-positive dopaminergic neurons in POLG^Q811R^ MDNS.

To further understand the biological relevance of our data, we performed Ingenuity Pathway Analysis (IPA) to identify key regulated pathways corresponding to the protein changes identified in POLG^Q8111R^ MDNS, and IPA revealed several altered canonical pathways (Fig. 4g). Notably, two of the up-regulated proteins in the in POLG1^Q811R^ MDNS, MARK11 and ARG2, appeared to be linked to several pathways, including Parkinson’s signaling, IL-10 signaling, AMPK-signaling, HIF-alpha signaling, and pathways associated with urea cycle. MAPK11, also known as p38b, belongs to a family of p38 mitogen activated protein kinases (MAPK) [6]. MAPK has been implicated in induction of microglia activation, oxidative stress, neuroinflammation and apoptosis in PD [33]. Cytoplasmic arginase 1 and mitochondrial arginase 2 are responsible for converting L-arginine to L-ornithine and urea, and increased *ARG2* expression has been observed in a patient brain diagnosed with Alzheimer’s disease (AD) [27].

Disease and function analysis of the significantly altered proteins also indicated that POLG^Q811R^ was associated with alterations in pathways related to cell death and survival, organismal injuries and abnormalities, carbohydrate metabolism, cellular movements, cellular development, cellular growth and cellular proliferation (Fig. 4h).

### Increased pigmentation of dopaminergic neurons in POLG1^Q811R^ variant MDNS may be linked to proteome alterations

**Fig. 5:**
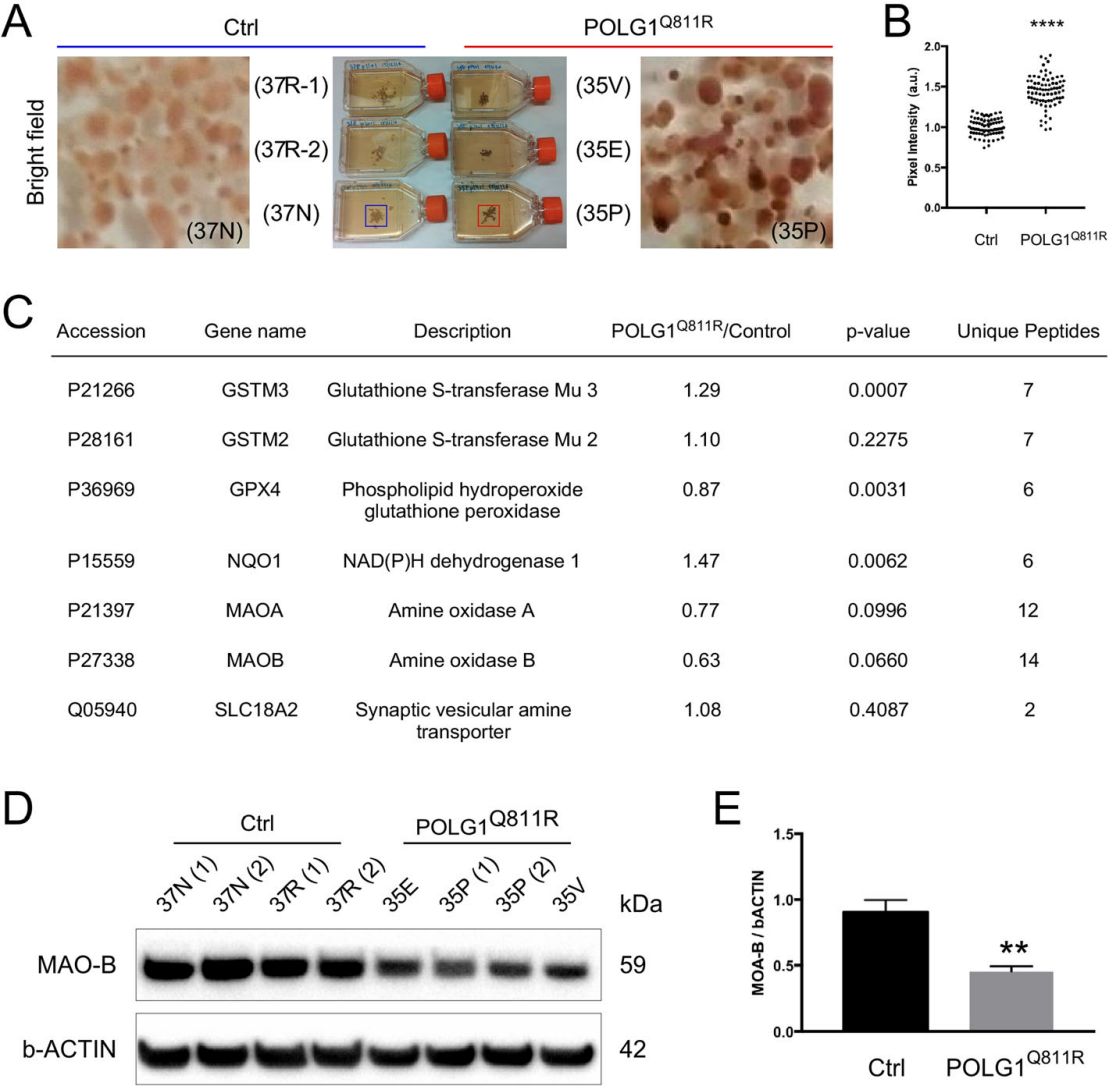
Increased neuromelanin production in POLG^Q811R^ variant MDNS may be linked to proteome alterations. **a** and **b** Bright field images showing the formation of NM-like pigmented granules in *POLG1* variant and healthy control MDNS. Quantification of the pigmentation of the MDNS in a.u. Results are presented as ± SEM; *n* = 80 MDNS counted; **** represents *P* < 0.0001; unpaired *t*-test. **c** Abundance levels for proteins linked to neuromelanin production in *POLG1* variant and healthy control MDNS identified by LC MS/MS quantitative proteomics. **d** and **e** Western blot showing a significant decrease in levels of MAO-B in the *POLG1* variant compared to healthy control MDNS. Quantification of MAO-B levels are shown normalized to b-ACTIN. Results are presented as ± SEM; n = 4 independent experiments; ** indicates *P* < 0.01; unpaired *t*-test

In accordance with previous observations [35], both control and POLG1^Q811R^ MDNS exhibited dark-pigmented deposits suggestive of accumulation of neuromelanin (NM) in dopaminergic neurons (Fig. 5a). NM biosynthesis is involved in regulating levels of cytosolic DA and DA quinones [71]. The level of pigmentation was higher for POLG1^Q811R^ MDNS than control MDNS (Fig. 5a and b). Cytosolic DA can oxidize to form aminochrome, a precursor to NM that can form adducts with proteins involved in pathogenesis of PD, including aSYN, and has been shown to mediate mitochondrial and lysosomal dysfunction in PD [14, 66]. Besides NM formation, DA oxidation can be prevented by degradation of excess cytosolic DA by monoamine oxidase (MAO) located on the outer mitochondrial membrane or by the uptake of DA into synaptic vesicles by VMAT2 [49]. Additionally, to avoid neurotoxicity, aminochrome may undergo two-electron reduction by NQO1 to leukoaminochrome or conjugation with glutathione to offer neuroprotection [64, 65]. Therefore, we assessed levels of MAO-A, MAO-B, VMAT2, NQO1, and glutathione S-transferase M2–2 responsible for conjugation of aminochrome with glutathione. We also examined the level of glutathione peroxidase 4 GPX4, that has been found to colocalize with NM and is significantly reduced in the *SNpc* of PD patients, and which is increased when cell loss occurs [8].

Quantitative proteomic analysis showed no significant alterations in these proteins based on the *P* value of 0.001 chosen for significance (Fig. 5c); however, NQO1 showed trends towards upregulation in POLG1^Q811R^ MDNS. GPX4, known to colocalize with neuromelanin showed a trend towards downregulation, in line with the reduction observed in *SNpc* of PD patients [8]. Interestingly, another glutathione S-transferase isoform - GSTM3, was significantly upregulated in the POLG1^Q811R^ MDNS, compare to control (Fig. 5c). Although little is known about the role of GSTM3 in aminochrome metabolism, upregulation of GSTM3 in POLG1^Q811R^ cultures may play a part in protection against aminochrome-related oxidative stress [5]. Proteome levels of MAO-A and MAO-B in the POLG^Q811R^ MDNS showed a trend towards a decrease. In line with that, Western blot analysis of MAO-B levels revealed significant decrease in POLG1^Q811R^ MDNS compared to control samples (Fig. 5d, e). Taken together, these data suggest that increased pigmentation, suggestive of NM-accumulation in the patient iPSC-derived brain dopaminergic neurons, could be due to decreased DA degradation under the POLG1^Q811R^ MDNS condition due to the additive effect of lower levels of MAO-A and MAO-B.

### Western blot analysis reveals comparable levels of mitochondria-associated proteins between healthy and POLG1^Q811R^ MDNS

**Fig. 6:**
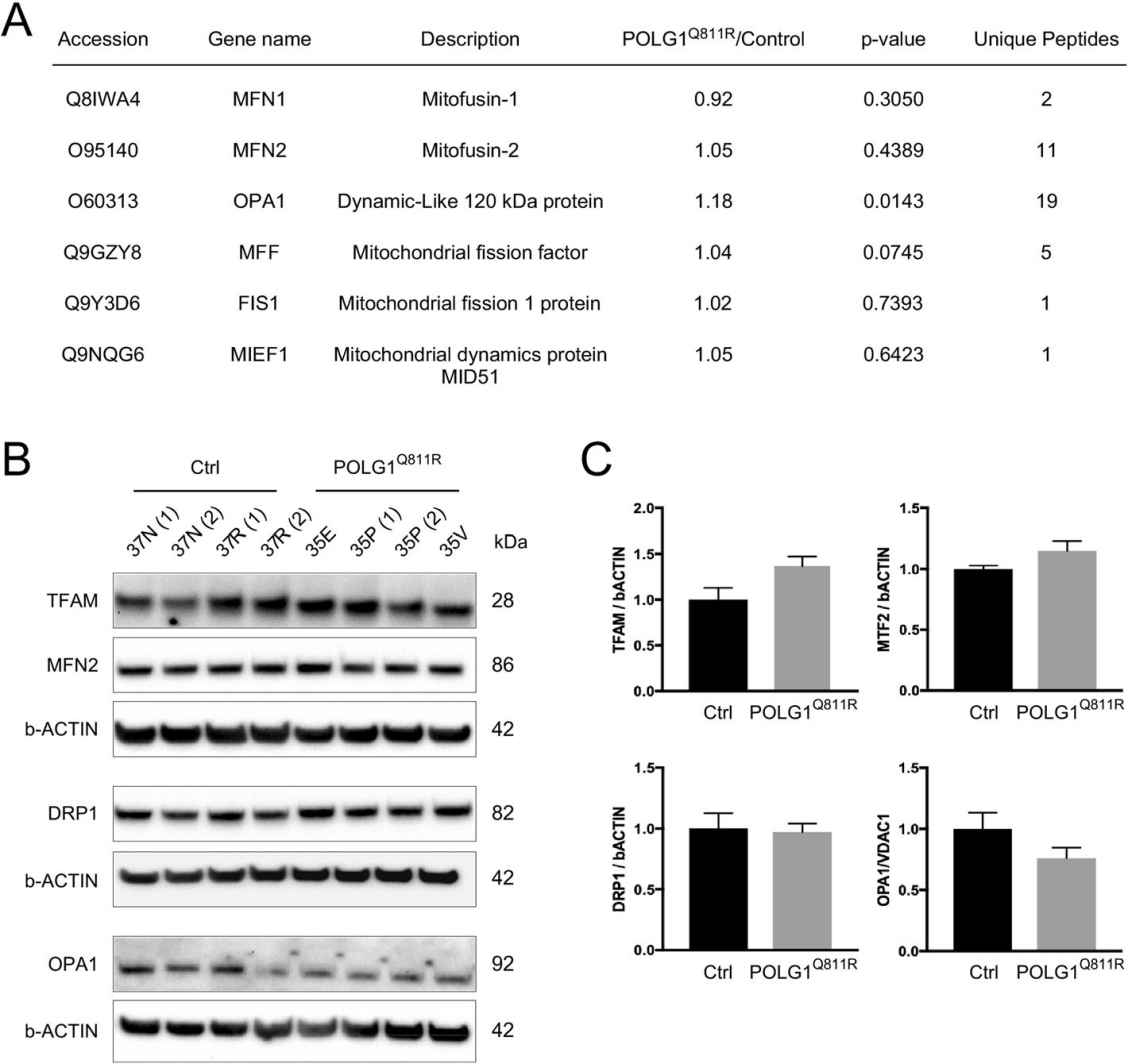
Western blot analysis reveals comparable levels of mitochondria-associated proteins between healthy and POLG^Q811R^ variant MDNS. **a** Abundance levels for mitochondrial markers MFN1, MFN2 and OPA1 involved in mitochondrial fusion, and MFF, FIS1 and MIEF1 regulating mitochondrial fission in *POLG1* variant and healthy control MDNS, identified by LC MS/MS quantitative proteomics. **b** Western blot analysis confirms no difference in expression levels of mitochondrial transcription factor TFAM, fusion-related OPA1 and MFN2 and fission-related DRP1 in *POLG1* variant and healthy control MDNS. **c** Quantification of protein levels from (**b**), normalized to b-ACTIN. Results are presented as ± SEM; n = 4 independent experiments; unpaired *t*-test

In addition to POLG1, several other mitochondria-associated proteins are known to be involved in the maintenance of mitochondrial integrity and they may be affected in PD [13]. Therefore, we assessed the levels of several mitochondria-associated proteins involved in mitochondrial function, including mitochondrial transcription factor A (TFAM) essential for mitochondrial DNA transcription [20, 42], as well as several mitochondrial proteins involved in regulation of mitochondrial dynamics through fusion and fission [53, 62]. Proteomics analysis (Fig. 6a) and Western blot analysis (Fig. 6b and c) confirmed no significant changes for mitochondria dynamics-associated proteins, between POLG1^Q811R^ MDNS and control samples.

### Functional changes in glycolysis but not in the oxidative phosphorylation of POLG1^Q811R^ cultures support protein alterations identified by quantitative proteomic analysis

**Fig. 7:**
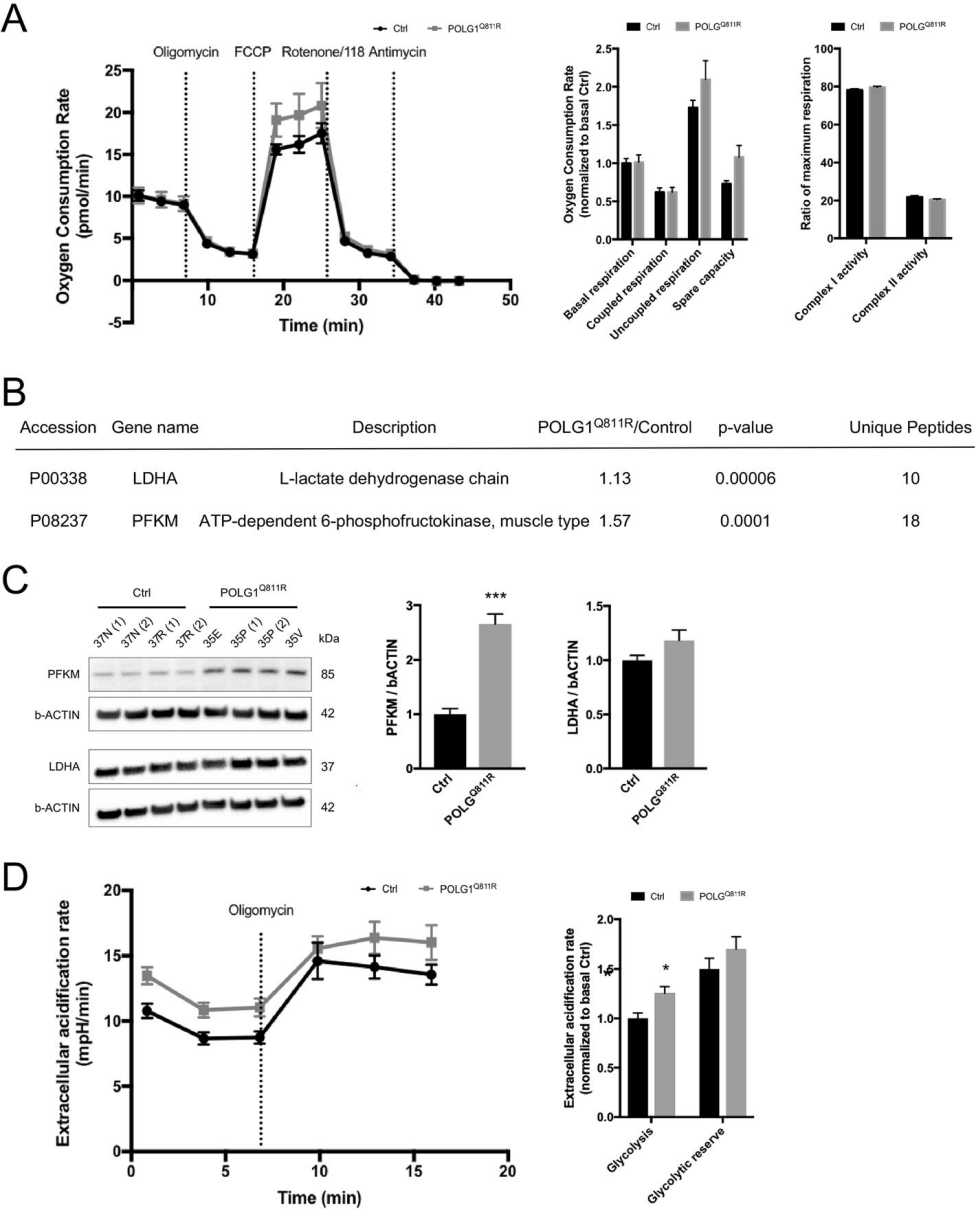
Functional changes in glycolysis but not in the oxidative phosphorylation of POLG1 p.Q811R variant cultures support protein alteration identified by quantitative proteomic analysis. **a** Oxygen consumption rates (OCRs) of 100 DIV dissociated *POLG1* variant and healthy control cultures, normalized to protein content, illustrating levels of basal respiration, ATP-coupled (oligomycin-sensitive) respiration, ATP-uncoupled respiration after FCCP treatment, spare respiratory capacity, as well as contribution of mitochondrial complex I- and complex II-linked respiration calculated from the effect of treatment with NV118, a cell-permeable prodrug of the complex II substrate succinate. Results are presented as ± SEM, n = 4 independent experiments; unpaired *t*-test. **b** Abundance levels for LDHA and PFKM in *POLG1* variant and healthy control MDNS identified by LC MS/MS quantitative proteomics. **c** Western blot analysis shows significant upregulation of PFKM and a trend for increase of LDHA in POLG^Q811R^ MDNS compared to healthy control MDNS. Protein levels are normalized to b-ACTIN. Results are presented as ± SEM; n = 4 independent experiments; *** indicates *P* < 0.001; unpaired *t*-test. **d** Extracellular acidification rates (ECARs) of 100 DIV dissociated *POLG1* variant and healthy control cultures, normalized to protein content, illustrating levels of basal glycolysis and glycolytic reserve following treatment with oligomycin. Results are presented as ± SEM n = 4 independent experiments. * indicates *P* < 0.05; unpaired *t*-test

IPA of significantly altered proteins revealed several pathways associated with energy metabolism, including 2-ketoglutarate dehydrogenase complex, pyruvate fermentation to lactate, AMPK signaling and HIF1-alpha signaling. Therefore, we decided to evaluate rates of mitochondrial respiration and glycolysis in POLG1^Q811R^ and control cultures. First, we assessed oxygen consumption rates (OCR) in POLG1^Q811R^ and control cultures using Seahorse XF Cell Mito Stress Test Kit. There were no significant changes in basal and ATP coupled and uncoupled respiration between POLG1^Q811R^ and control cultures (Fig. 7a); moreover, the contributions of complex I and II of the respiratory chain towards maximum respiration were comparable. In line with that, proteomic analysis revealed no changes in proteins associated with electron transport chain complexes I-IV (Additional file 3: Figure S2). Proteomic analysis identified two significantly upregulated enzymes in POLG^Q811R^ MDNS, lactate dehydrogenase A (LDHA) and phosphofructokinase, muscle type (PFKM), that are both involved in glycolysis (Fig. 7b) [81]. Western blot confirmed significantly elevated levels of PFKM, a rate limiting enzyme of glycolysis, but did not show any significant change in the levels of LDHA, although there was a trend for increase (Fig. 7c). The discrepancy between Western blot and proteomics data for LDHA levels could be due to the low sensitivity of the Western blot, as based on proteomic analysis, LDHA had a very moderate increase (Fold change = 1.13 with *P* value of 0.00006). We therefore decided to examine the extracellular acidification rates (ECAR) from our Mito Stress experiments. ECAR, indicative of lactic acid production in glycolysis showed a significant increase in basal glycolysis within POLG1^Q811R^ cultures, compared to control cultures (Fig. 7d). In a similar manner, elevated aerobic glycolysis is observed in many cancers [24], and many cancers are associated with elevated expression of LDHA, responsible for conversion of pyruvate to lactate at the last step of glycolysis and regulated by HIF1-alpha signaling amongst others [22]. LDHA was also upregulated in POLG1^Q811R^ cultures, and upregulation of LDHA gene has been reported in PD patients [39].

**Fig. 8:**
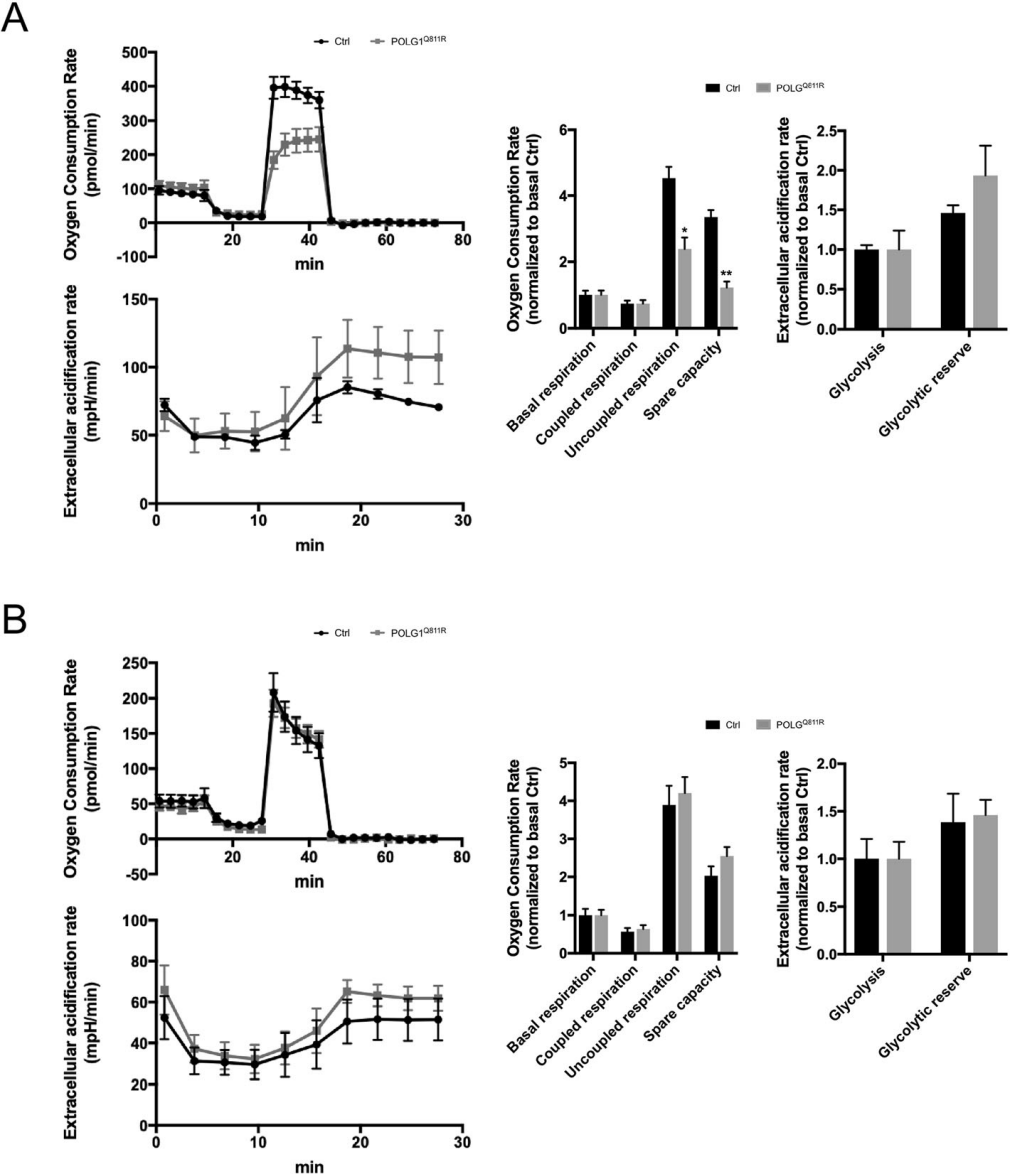
Oxygen consumption and extracellular acidification rates of control and POLG1 p.Q811R variant fibroblasts and iPSC-derived astrocytes. **a** Oxygen consumption rates (OCRs) and extracellular acidification rates (ECARs) of 100 DIV *POLG1* variant and healthy control astrocytes, normalized to protein content. OCR was used to calculate basal respiration, ATP-coupled and uncoupled respiration and spare respiratory capacity; glycolysis and glycolytic reserve were calculated from ECAR. Results are presented as ± SEM, *n* = 3 independent experiments; * indicates *P* < 0.05; ** indicates P < 0.01; unpaired *t*-test. **b** Oxygen consumption rates (OCRs) and extracellular acidification rates (ECARs) of cultured fibroblasts from healthy control and POLG^Q811R^ patients, normalized to protein content. OCR was used to calculate basal respiration, ATP-coupled and uncoupled respiration and spare respiratory capacity; glycolysis and glycolytic reserve were calculated from ECAR. Results are presented as ± SEM, n = 3 independent experiments; unpaired *t*-test

Since our MDNS are not purely neuronal cultures, but also contain a portion of astrocytes (Fig. 2b and c), which are known to be highly glycolytic [7] we then performed the same analysis on homogenous cultures of iPSC-derived astrocytes from control and POLG^Q811R^ lines. We also assessed the OCR and ECAR in parental fibroblasts to examine if the same phenotype could also be identified. In contrast to our observations from MDNS, astrocytes did not show any alterations in glycolysis, but exhibited decreased rate of uncoupled respiration, and mitochondrial spare capacity (Fig. 8a). Fibroblasts from control and POLG^Q811R^ patient did not show any disease phenotype in metabolic rates (Fig. 8b). Taken together, these results suggest that the elevated glycolysis in POLG^Q811R^ MDNS may be attributed to the neurons.

## Discussion

Neurodegeneration of midbrain dopaminergic neurons is a key hallmark of PD. Using reprogramming technologies, it is possible to generate patient-specific iPSCs, and further differentiate them into brain cell types, opening up the possibility to assess early cellular alterations associated with a patient specific neuropathology. Here, we report a case study of a female PD patient with a p.Q811R (c.2432A > G) variation in the polymerase domain of the POLG1 gene. The patient presented cataract, early-onset parkinsonism, premature ovarian failure and sign of fatigue and myopathy, resulting in the final diagnosis of adPEO.

In order to investigate her parkinsonian phenotype on a cellular level, we employed a recently developed protocol to obtain iPSCs-derived pigmented dopaminergic neurons-containing MDNS. The MDNS were predominantly composed of MAP2-positive neurons. They had a midbrain dopaminergic identity, confirmed by co-expression of VMAT2, FOXA2 and TH (Fig. 2b) [41]. Astrocytes (GFAP-positive), whose role in PD has recently emerged [12], were also detected. Prior to the read-outs, the MDNS were differentiated for 100 DIV. Several recent studies showed that maintaining iPSC-derived neural cultures beyond 100 days allows cells to fully mature [67], and leads to the emergence of PD phenotypes including DA oxidation mediating mitochondrial and lysosomal dysfunction [14, 16, 46].

Quantitative proteomic analysis of MDNS yielded 61 differentially expressed proteins between POLG1^Q811R^ and control MDNS (Fig. 4b and c). Interestingly, the IPA analysis identified several key pathways in POLG1^Q811R^ linked to increased neuroprotection and neuronal survival, which could explain the higher number of surviving dopaminergic neurons following dissociation of the MDNS. The MAPK11 protein that was upregulated in POLG1^Q811R^ MDNS compared to control MDNS has been linked to several canonical pathways by IPA, involving p38 MAPK signaling (Fig. 4g). P38 MAPK activation has been shown to contribute to PD pathology by driving neuroinflammation and neurodegeneration through activation of microglia and induction of NO production [75]. Interestingly, extracellular NM activates p38 MAPK pathway, also leading to microglial activation [75]. Mitochondrial enzyme ARG2 was also upregulated in POLG1^Q811R^ MDNS, implicating urea cycle-related pathways of arginine degradation, citrulline biosynthesis and metabolism (Fig. 4g). A recent study identified increased ARG2 in AD patient brain [27]. ARG2 is thought to have a neuroprotective function due to its negative effect on NO production [3]. However, NO, known to lead to neurotoxicity when at high levels, is also important for synaptic plasticity, and the observations of altered arginine metabolism in AD brain also opens the question whether ARG2 increase is a consequence or a cause in AD pathogenesis, and its implication in the progression of the disease [43]. The role of the urea cycle and arginine metabolism in PD have not been investigated yet, and could not be ruled out as potential new mechanisms involved in PD pathogenesis.

Addition of DA during maturation stages of the differentiation, triggered the darkening of the neurons that composed the MDNS (Fig. 5a), suggesting higher levels of NM in POLG1^Q811R^ neurons [35]. NM is an insoluble pigment found in catecholaminergic neurons of the SN and some other brain regions, believed to protect neurons from neurotoxic DA quinones and iron, but that may also increase neuronal vulnerability during neurodegeneration [82]. However, the exact function and involvement of NM in PD is still not fully understood. The proteomic data together with the stronger pigmentation observed was suggestive of higher NM production/accumulation or defect in NM degradation in the POLG1^Q811R^ MDNS compared to controls (Fig. 5a). Indeed, proteomic analysis revealed a trend towards decreased levels of DA degrading enzymes, MAO-A and MAO-B, and a significant decrease of MAO-B measured by Western blotting (Fig. 5d and e). However, PD pathology is normally associated with increased MAO-B activity, and MAO-B inhibitors are effective in the initial treatment of PD [38]. Our data therefore raises the question whether PD dopaminergic neurons are prone to accumulate NM very early in the disease, compare to control dopaminergic neurons. A possible explanation could be that decrease in MAO-A and MAO-B levels was prompted by the supplementation of the differentiation media with DA. It has been reported that chronic treatment with L-DOPA leads to inhibition of MAO activity, an effect opposite of that of acute L-DOPA treatment [57]. However, a similar inhibitory effect would then be expected in the control MDNS. It is also possible that the decrease in MAO-B is a protective mechanism against mitochondrial pathology in POLG1^Q811R^ MDNS, as the activity of the enzyme has been linked to increased ROS and mitochondrial impairment [37, 70]. Notably, despite PD therapies focusing predominantly on inhibition of MAO-B to reduce DA breakdown and, potentially, to decrease formation of toxic DA oxidation products [77, 78], the MAO-B isoform is predominantly expressed in glial cells, while the DA breakdown in neurons occurs mainly via the MAO-A isoform [23]. A recent study assessed expression levels of MAO-A and MAO-B in different brain regions in patient with PD and PD-associated disorders, postulating that elevated MAO-B could be a marker of astrogliosis [73]. Reportedly, elevated MAO-B was observed in the *SNpc* of patients with progressive supranuclear palsy and, to some extent, MSA, but no significant changes were detected in PD patients, consistent with previous reports of limited astrogliosis in PD [72]. On the other hand, transcriptional upregulation of MAO-A and MAO-B have been reported in patients with PARK2 and GBA variants in iPSC-derived neurons-based studies [34, 76]. Employing a differentiation protocol that includes addition of L-DOPA or DA may help in the future to examine NM formation and DA metabolism in PD neurons, in vitro.

Alteration in POLG1^Q811R^ MDNS energy metabolism was identified by the proteomic analysis and IPA (Fig. 4), and confirmed by the increased glycolysis rate, but not the oxidative phosphorylation in POLG1^Q811R^ MDNS, compared to controls (Fig. 7). Elevated glycolysis rate was in line with the upregulation of PFKM essential for the drive of glycolysis and LDHA that is responsible for the conversion of pyruvate to lactate. Upregulation of genes encoding LDHA, have been observed in animal models of PD and samples from PD patients, and has been associated with aging and increased mtDNA mutations, thought to upregulate glycolysis as a compensatory mechanism for a decrease in ATP production [39, 58, 60]. A study in mice carrying mtDNA mutations expressing a proofreading-deficient version of *POLG1* reported elevated brain levels of lactate due to an increase in LDHA/LDHB ratio [58]. Additionally, a recent study in a transgenic PS1/APP AD mouse model revealed decreased content of lactate as well as downregulation of LDHA and LDHB in PS1/APP mice, but an increase in LDHA/LDHB ratio postulated to compensate for neuronal lactate deficit and increase lactate production [80]. It has also been shown that increase in LDHA can mediate resistance to Aβ toxicity through upregulation of aerobic glycolysis as a protective mechanism, but it is possible that this effect is present in the prodromal stages of AD [50], while at a later stages of the disease aerobic glycolysis and elevated lactate production may contribute to the cognitive decline associated with AD [28]. Notably, the elevated levels of LDHA in skeletal muscle have also been reported in cohorts of patients with PEO and mitochondrial encephalopathy lactic acidosis and stroke-like episodes syndrome (MELAS) [61], suggesting implications for not only PD, but also mitochondrial diseases.

The implications of elevated glycolysis in PD is somewhat controversial. Mitochondrial dysfunction and increased glycolysis has been observed in the peripheral blood of prodromal and early-stage PD patients [68]. In addition to that, PARK2 and PINK1, loss-of-function variations of which lead to familial PD, have recently been shown to negatively regulate the Warburg effect, a switch from mitochondrial respiration to aerobic glycolysis when oxygen supply is normal in cancer [2, 79]. These results implicate increased glycolysis in the pathogenesis of PD. At the same time, two recent studies showed neuroprotective effect of glycolysis-enhancing drugs Meclizine and Terazosin, suggesting that glycolysis can provide beneficial effect when energy metabolism and ATP production is compromised [15, 32].

Our iPSCs-based model, for investigating neuronal phenotype and pathology in POLG^Q811R^ variant with early-onset PD, is a valuable tool to gain insights into cellular mechanisms affected in this patient (catecholamine degradation, aSYN aggregation, energy metabolism) and to establish a link between clinical and cellular phenotypes.

## Materials and methods

### Generation and maintenance of iPSCs

Human dermal fibroblasts were obtained by punch skin biopsy from a PD patient carrying heterozygous p.Q811R variation in the POLG1 gene (POLG1^Q811R/WT^; referred to as POLG1^Q811R^ in the text and figures), after written informed consent. Fibroblasts were cultured and expanded in culture medium containing DMEM media (Thermo Fisher Scientific), 10% fetal bovine serum and 1% Penicillin-Streptomycin. For reprogramming, fibroblasts were transduced using CytoTune™-iPS 2.0 Sendai reprogramming kit (Thermo Fisher Scientific) according to manufacturer’s instructions. The cells were maintained in fibroblast media with daily media changes until day 6, when the cells were re-seeded onto a layer of irradiated mouse embryonic fibroblasts (MEF) feeder cells in WiCell medium composed of advanced DMEM/F12, 10% Knock-Out Serum Replacement, 2 mM L-glutamine, 1% non-essential amino acids (all from Thermo Fisher Scientific), 50 μM β-mercaptoethanol (Sigma-Aldrich) and 20 ng/ml FGF2 (Thermo Fisher Scientific). Three to four weeks later, individual colonies were picked and expanded, before being bio-banked and characterized, similarly to our previous lines [31].

### Differentiation of iPSCs towards midbrain dopaminergic identity

To differentiate the iPSC into midbrain dopaminergic neurons, we modified previously established protocols [35] was used. Briefly, following expansion, iPSCs were detached with dispase II (Thermo Fisher Scientific) and seeded in ultra-low attachment flasks in WiCell media supplemented with 10 mM ROCK inhibitor Y27632 (Sellekchem) and 20 ng/ml FGF2 (Thermo Fisher Scientific). The following day (denoted as day 0), the media was changed to neural induction medium (NIM) composed of advanced DMEM/F12, 2 mM L-glutamine, 1% non-essential amino acids, 1% N2 supplement, 1% Penicillin-Streptomycin (all Thermo Fisher Scientific). Media was supplemented with 0.1 μM LDN (Stemgent), 10 μM SB 431542 (Sigma-Aldrich), 200 ng/ml SHH-C (Thermo Fisher Scientific), 1 μM SAG (Sellekchem) and 0.8 μM CHIR (Sigma-Aldrich) (day 0–4) and replaced every other day. On day 6 SB and SHH-C were removed from the media and SAG was increased to 2 μM, and on day 10 LDN was removed also. From day 12 onwards the cells were grown in NIM supplemented with 100 ng/ml FGF8 (Thermo Fisher Scientific) and 2 μM SAG, 10 ng/ml BDNF (R&D Systems) and 200 μM AA (Sigma-Aldrich). From day 22, the media was replaced with neural differentiation medium (NDM) containing Neurobasal media with 2 mM L-glutamine, 1% non-essential amino acids, 1% N2 supplement, 1% B27 without vitamin A, 1% Penicillin-Streptomycin (all from Thermo Fisher Scientific), supplemented with 100 ng/ml FGF8 and 2 μM SAG, 10 ng/ml BDNF, 10 ng/ml GDNF (R&D Systems), 200 μM AA, 500 μM db-cAMP (Sigma-Aldrich) and 1 ng/ml TGFb (Peprotech). From day 30 onwards, FGF8 and SAG were removed from the media and 50 μM DA (Sigma-Aldrich) was added to the media to promote formation of neuromelanin. For the readouts, differentiated spheroids at 100 DIV were either collected, washed and snap-frozen or fixed, for analysis of the proteome, and immunocytochemistry (ICC), respectively, or dissociated using 0.05% Trypsin-EDTA (Thermo Fisher Scientific) and re-seeded on plates coated with 40 μg/ml poly-ornithine (Sigma-Aldrich) and 15 μg/ml laminin (Thermo Fisher Scientific), ICC, amperometry and SeaHorse analysis.

### Differentiation of iPSCs towards astrocytes

The differentiation of healthy and POLG^Q811R^ iPSCs into astrocytes followed the same protocol as for midbrain dopaminergic identity, for the first 10 days. From day 12, cells were differentiated in NIM media supplemented with 100 ng/ml FGF8 and 2 μM SAG until day 30. Then, cells were dissociated, filter-strained (100 μm), and transferred to new ultra-low-adherent flasks with neural expansion medium (NEM) containing DMEM-F12, 2 mM L-glutamine, 1% non-essential amino acids, 1% N2 supplement, 1% B27 without vitamin A, 1% Penicillin-Streptomycin, and 0.2 μg/ml heparin (Sigma-Aldrich), supplemented with 20 ng/ml FGF2 and 20 ng/ml EGF (Peprotech). On day 60, spheroids were washed and dissociated into single cells, and seeded to adherent culture flasks coated with Poly-L-ornithine/laminin, in NEM containing 20 ng/ml CNTF (R&D Systems). Finally, from day 80 onwards, astrocytes were cultured in NDM with 20 ng/ml CNTF. Cells were passaged at confluency.

### Amperometry

In vitro high-speed chronoamperometric measurements (2 HZ) of DA release, were performed according to previously described protocol [4, 29], on dissociated spheroids seeded onto 4 well-plates at a density of about 80,000 cells/cm^2^. Briefly, Recording was done using FAST-16 mk III hardware (Quanteon) coupled to Nafion®-coated carbon fiber electrodes (30 μm diameter, 150 μm length). A square wave potential of 0.55 V; 0.0 V resting potential was applied vs an Ag/AgCl reference. Prior to recording the electrode was calibrated at room temperature in stirred 0.1 M PBS by on addition of Ascorbic Acid (20 mM) followed by three additions of DA (2 μM). The electrodes used displayed a linearity correlation of > 0.99, a selectivity of DA over ascorbic acid of > 1000:1, and a limit of detection below 0.01 μM.

### Immunocytochemisty

For ICC differentiated spheroids were either collected or dissociated at 100 DIV and seeded onto poly-ornithine/ laminin-coated clear bottom 96-well microplates (Greiner Bio One) at 200,000 cells/cm^2^ and fixed in 4% paraformaldehyde (Sigma-Aldrich). After fixation cells were washed in PBS, and fixed spheroids were equilibrated in 30% sucrose overnight. Fixed spheroids were then mounted in OCT (Sigma-Aldrich) and cut using a cryostat into 20 μM sections. Dissociated cells and spheroid sections were blocked in 10% donkey serum in PBS with 0.1% (dissociated cells) PBS-Tween 20 with or 0.3% (sections) Triton-X (Sigma-Aldrich). Primary antibodies were diluted in blocking solution and incubated overnight at 4 °C: mouse anti-AFP (Sigma-Aldrich, A8452, 1:500), mouse anti-SMA (Sigma-Aldrich, A2547, 1:500), rabbit anti-TUJ1 (Covance, PRB-435P, 1:500), mouse anti-Oct4 (Millipore, MAB4401, 1:200), mouse anti-Nanog (BD Biosciences, 560,483, 1:200), mouse anti-TRA-1-81 (Thermo Fisher Scientific, 411,100, 1:200), rabbit anti-TH (Millipore, AB152, 1:500), mouse anti-TH (Millipore, MAB318, 1:250), sheep anti-TH (Abcam, ab113, 1:500), goat anti-FOXA2(Santa-Cruz, sc11415, 1:250), rabbit anti-VMAT2 (Immunostar, 20,042, 1:500), mouse anti-aSYN (Santa-Cruz, sc12767, 1:200), rabbit anti-GFAP (DAKO, Z0334, 1:2000), chicken anti-MAP2 (Abcam, ab92434, 1:2000). AlexaFluor-488, AlexaFluor-555 and AlexaFluor-647-labelled secondary antibodies (Thermo Fisher Scientific) were used at 1:400 in PBS or PBS-Tween 20 at RT for 1 h. DAPI (1:10,000) was used for nuclei counterstaining. Images were acquired using an inverted epifluorescence microscope LRI-Olympus IX-73, Metamorph and ImageJ software were used for image analysis and quantification.

### Western blot

Protein extraction was performed using M-PER (Thermo Fisher Scientific) following manufacturer’s instructions. Pierce BCA Protein Assay Kit (Thermo Fisher Scientific) was used to quantify the protein. 13 μg of protein from each sample was loaded on Bolt 4–12% Bis-Tris Plus Gels (Thermo Fisher Scientific) and then transferred to nitrocellulose membranes using iBlot transfer device (Thermo Fisher Scientific). Membranes were blocked in with either 5% skim milk (Sigma-Aldrich) or 5% BSA (for phosphorylated protein) (VWR) diluted in PBS-Tween 20. The membranes were incubated with primary antibodies diluted in blocking solution at 4 °C, overnight: mouse anti-Actin (Sigma-Aldrich, A5441, 1:20000), rabbit anti-ASYN (Cell Signaling, 2642S, 1:1000), mouse anti-ASyO5 (Agriseria, AS132718, 1:1000), rabbit anti-phosphoS129 (Abcam, ab168381, 1:500), mouse anti-DRP1 (Abcam, ab56788, 1:500), rabbit anti-MAO-B (Abcam, ab175136, 1:2000), mouse anti-MFN2 (Abcam, ab56889, 1:500), anti-rabbit anti-OPA1 (Abcam, ab157457, 1:500), rabbit anti-TH (Millipore, AB152, 1:2000), mouse anti-TH (Millipore, MAB318, 1:1000), mouse anti-TOMM20 (Abcam, ab56783), rabbit anti-TOMM20 (Santa Cruz, sc11415, 1:200), rabbit anti-TFAM (Abcam, ab131607, 1.200), rabbit anti-VDAC1 (Abcam, ab15895, 1:1000), rabbit anti-LDHA (Cell Signaling, #2012, 1:1000), rabbit anti-PFKM (Abcam, ab97353, 1:1000). Washed membranes were incubated with peroxidase-conjugated secondary antibody (R&D Systems) at 1:2000 and developed using ChemiDoc gel imaging system (Bio-Rad). Analysis of the blots was performed using Bio-Rad Image Lab software and the protein levels were normalized to beta actin.

### Oxygen consumption rate

Control and POLG^Q811R^ MDNS dissociated cultures, astrocytes and fibroblasts were seeded onto poly-ornithine/laminin-coated Seahorse 96-well plates (Agilent Technologies) at 200,000 to 300,000 cells/cm^2^. Empty wells were used as background controls. Oxygen consumption rates (OCR) and extracellular acidification rate (ECAR) analysis was performed 2 days later, on the Seahorse XFe 96 analyzer using the Seahorse XF Mito Stress Test Kit (Agilent Technologies), according to manufacturer’s instructions. Briefly, cells were washed three times and incubated in XF-Base medium supplemented with 5 mM pyruvate, 2 mM L-Glutamine and 10 mM glucose in non-ventilated, non-CO_2_ incubator at 37 °C for 1 h. For measurements, three cycles for MDNS dissociated cultures and 5 cycles for astrocytes and fibroblasts of 30 s mix, followed by 2 min measurement were used for the baseline and sequential treatments of 1 μg/mL Oligomycin, 1 μM FCCP, 2uM Rotenone (for MDNS, rotenone was combined with 500 μM succinate prodrug NV118), 1 μg/mL Antimycin. Background-corrected measurements of OCR and ECAR were normalized to the total protein level using Protein Assay Kit II (Bio-Rad).

### LC MS/MS proteomics

#### Protein extraction for proteomic analysis

Cell pellets were homogenized using a FastPrep®-24 instrument (MP Biomedicals, www.mpbio.com) with Lysing Matrix D for five repeated cycles (speed 6.5 m/s, 40 s/cycle) in 200 μl of the buffer containing 2% sodium dodecyl sulfate and 50 mM triethylammonium bicarbonate (TEAB). Samples were centrifuged at 16000 g for 10 min and the supernatants were transferred to clean tubes. The lysis tubes were washed with 100 μl of the lysis buffer, centrifuged at 16000 g for 10 min, the supernatants were combined with the corresponding lysates from the previous step. Protein concentration in the combined lysates was determined using Pierce™ BCA Protein Assay Kit (Thermo Scientific) and the Benchmark™ Plus microplate reader (BIO-RAD) with bovine serum albumin (BSA) solutions as standards. A representative reference sample was prepared, containing equal amounts from the 8 individual samples.

#### Tryptic digestion and tandem mass tag (TMT) labelling

Aliquots containing 30 μg of each sample including the reference were reduced with 100 mM DL-dithiothreitol (DTT) at 56 °C for 30 min. The reduced samples were processed using the modified filter-aided sample preparation (FASP) method (Wisniewski JR et al. Nat Methods. 2009 May;6(5):359–62). In short, reduced samples were transferred to 30 kDa MWCO Pall Nanosep centrifugation filters (Sigma-Aldrich) and washed twice with 8 M urea. Additional washes with digestion buffer (1% sodium deoxycholate in 50 mM TEAB) was performed before and after alkylation with 10 mM methyl methanethiosulfonate for 30 min at room temperature. Protein digestions were performed using Trypsin (Pierce MS grade) in digestion buffer, first with 0.3 μg Trypsin at 37 °C overnight followed by new addition of 0.3 μg trypsin and incubation t at 37 °C for 2 h. Produced tryptic peptides were collected by centrifugation and labelled using TMT 10-plex isobaric mass tagging reagents (Thermo Scientific) according to the manufacturer instructions. Labelled samples were combined and sodium deoxycholate was removed by acidification with 10% TFA.

The combined TMT-labeled sample was fractionated into 40 primary fractions by basic reversed-phase chromatography (bRP-LC) using a Dionex Ultimate 3000 UPLC system (Thermo Fischer Scientific). Peptide separations were performed using a reversed-phase XBridge BEH C18 column (3.5 μm, 3.0 × 150 mm, Waters Corporation) and a linear gradient from 3 to 40% solvent B over 17 min followed by an increase to 100% B over 5 min. Solvent A was 10 mM ammonium formate buffer at pH 10.00 and solvent B was 90% acetonitrile, 10% 10 mM ammonium formate at pH 10.00. The primary fractions were concatenated into 20 fractions (1 + 21, 2 + 22, … 20 + 40), evaporated and reconstituted in 15 μl of 3% acetonitrile, 0.2% formic acid for nLC-MS/MS analysis.

#### nLC-MS/MS

The fractions were analyzed on an orbitrap Fusion™ Lumos™ Tribrid™ mass spectrometer interfaced with Easy-nLC1200 liquid chromatography system (Thermo Fisher Scientific). Peptides were trapped on an Acclaim Pepmap 100 C18 trap column (100 μm × 2 cm, particle size 5 μm, Thermo Fischer Scientific) and separated on an in-house packed analytical column (75 μm × 30 cm, particle size 3 μm, Reprosil-Pur C18, Dr. Maisch) using a linear gradient from 5 to 33% B over 77 min followed by an increase to 100% B for 3 min, and 100% B for 10 min at a flow of 300 nL/min. Solvent A was 0.2% formic acid and solvent B was 80% acetonitrile, 0.2% formic acid. MS scans were performed at 120000 resolution, m/z range 375–1375. MS/MS analysis was performed in a data-dependent, with top speed cycle of 3 s for the most intense doubly or multiply charged precursor ions. Precursor ions were isolated in the quadrupole with a 0.7 m/z isolation window, with dynamic exclusion set to 10 ppm and duration of 45 s. Isolated precursor ions were subjected to collision induced dissociation (CID) at 35 collision energy with a maximum injection time of 50 ms. Produced MS2 fragment ions were detected in the ion trap followed by multinotch (simultaneous) isolation of the top 10 most abundant fragment ions for further fragmentation (MS3) by higher-energy collision dissociation (HCD) at 65% and detection in the Orbitrap at 50000 resolutions, m/z range 100–500.

#### Proteomic data analysis

Identification and relative quantification were performed using Proteome Discoverer version 2.2 (Thermo Fisher Scientific). The database search was performed using the Mascot search engine v. 2.5.1 (Matrix Science, London, UK) with MS peptide tolerance of 5 ppm and fragment ion tolerance of 0.6 Da. Tryptic peptides were accepted with 1 missed cleavage; methionine oxidation was set as a variable modification, cysteine methylthiolation, TMT-6 on lysine and peptide N-termini were set as fixed modifications. Percolator was used for PSM validation with the strict FDR threshold of 1%.

Quantification was performed in Proteome Discoverer 2.2. TMT reporter ions were identified in the MS3 HCD spectra with 3 mmu mass tolerance, and the TMT reporter intensity values for each sample were normalized within Proteome Discoverer 2.2 on the total peptide amount. Only the unique identified peptides were considered for the relative quantification.

#### Differential expression analysis

Differentially expressed proteins were identified by using unpaired t-test analysis with a *P*-value cut-off of 0.001.

The differentially expressed proteins identified from unpaired t-tests were visualized using a volcano plot, that displays log2-fold-change against -log(10) (*p*-value) from the t-test.

To graphically present the distribution of the differentially expressed proteins identified for the samples a heatmap plot was used after non-supervised hierarchical clustering, with a P-value cut-off of 0.001 log2 transformed data, using the R-package *gplots*.

Protein expression variances between groups are displayed as principal component analysis (PCA) to show similarities or differences among the samples belonging to the two different groups using the R-packages *prcomp* and *ggplot2* on log2 transformed data.

Statistical analyses were performed using the R software (version 3.6.0, https://www.r-project.org/, The R project, Vienna, Austria).

#### Ingenuity pathway analysis

To identify significant canonical pathways in which differentially expressed proteins were enriched, pathway enrichment analysis was conducted with the web-based pathway analysis tool IPA (Ingenuity, Systems, www.ingenuity.com, Redwood City, CA). Differentially expressed proteins were uploaded into IPA along with the protein identifiers, *p*-values and fold change values. A cut-off of a 0.001 P-value was used for the proteins to be included in the analyses. Each identifier was mapped to the Ingenuity knowledge base. Canonical pathway analysis identified the pathways, from the IPA library of canonical pathways, which were most significant to the input data set firstly using a ratio of the number of proteins from the dataset in a given pathway divided by the total number of molecules that make up the canonical pathway and secondly a Fisher’s exact test to assess the probability of the association to the canonical pathway.

#### Statistics

Statistical analysis was performed using Prism 7 software (GraphPad). Data are presented as mean ± standard error of the mean. Comparisons between control and diseased groups (*N* = 4 per group) were analyzed using an unpaired *t*-test. A value of *p* < 0.05 was considered to be statistically significant for all measurements, except for the proteomic analysis where *p* < 0.001 was considered to be statistically significant. To calculate the intensity of pigmentation of the MDNS, we measured the pixels value ranging from 0 (black) to 255 (white) on a grey scale, and presented the results as pixel values subtracted from the white value (255); up to 80 MDNS counted per group.

## Supplementary information

**Additional file 1: Table S1.** TMT-LC-MS raw data.

**Additional file 2: Figure S1.** Characterization of the differentiated MDNS. (A) Quantification of MAP2^+^, GFAP^+^ and TH^+^ cells relative to total number of DAPI-labeled cells, shown per differentiation. (B) FOXA2^+^, VMAT^+^ and aSYN^+^ cells relative to TH-labeled cells in POLG1 variant and healthy control cultures, shown per differentiation. (C) Proportion of MAP2^+^ and GFAP^+^ cells out of DAPI in control and POLG^Q811R^ dissociated cultures.

**Additional file 3: Figure S2.** Abundance levels of proteins associated with subunits of complex I-IV of mitochondrial respiratory chain.

## Data Availability

Material and data are available; proteomic data are provided as Additional file 1: Table S1.
