# Supplementary figures and images for: Cellular alterations identified in pluripotent stem cell-derived midbrain spheroids generated from a female patient with progressive external ophthalmoplegia and parkinsonism who carries a novel variation (p.Q811R) in the POLG1 gene
Source: Acta Neuropathol Commun. 2019 Dec 16;7:208. doi: 10.1186/s40478-019-0863-7 (PMC6916051; doi:10.1186/s40478-019-0863-7)

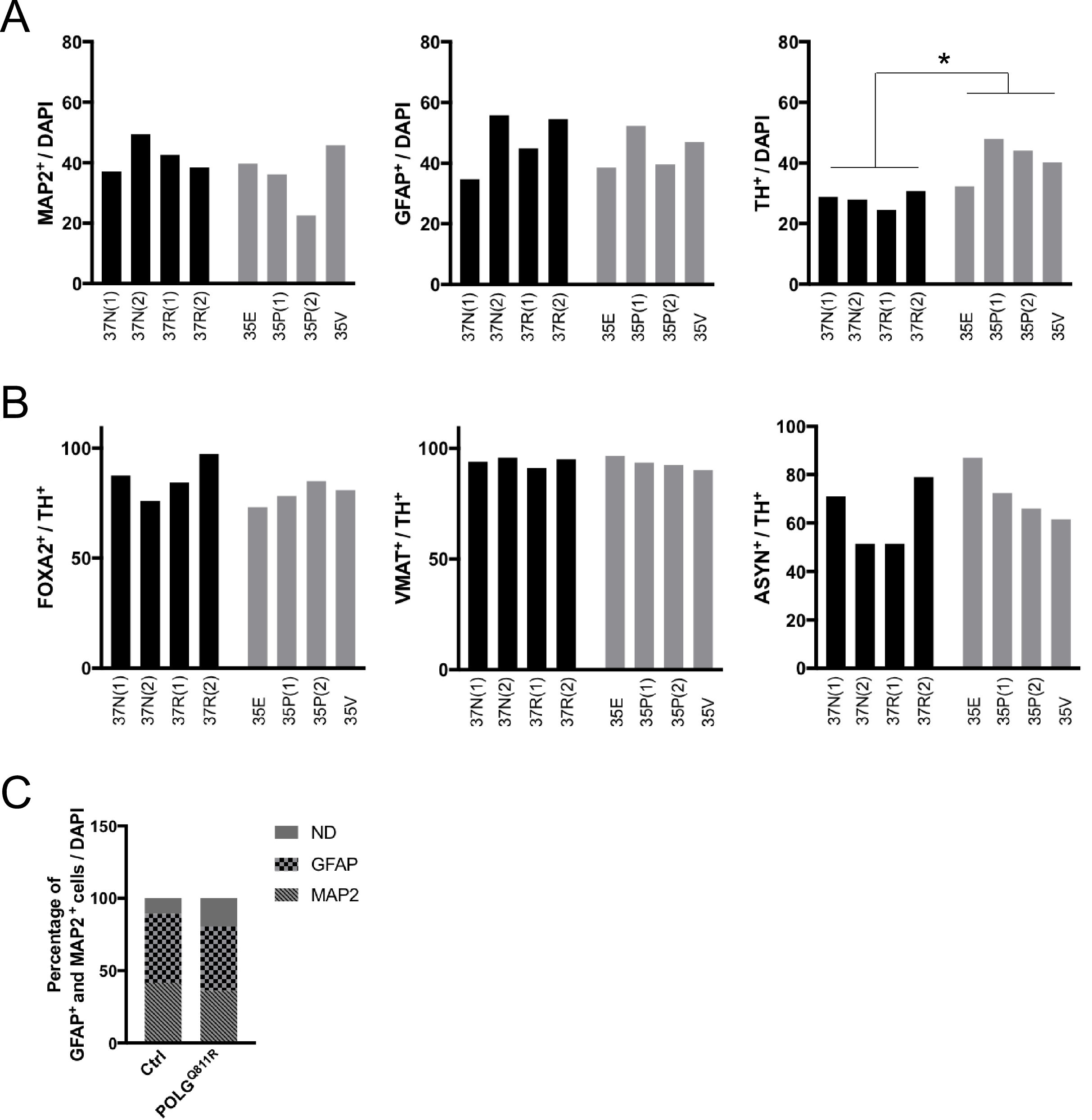

Supplement: Supplementary file 2 — Additional file 2: Figure S1. Characterization of the differentiated MDNS. (A) Quantification of MAP2+, GFAP+ and TH+ cells relative to total number of DAPI-labeled cells, shown per differentiation. (B) FOXA2+, VMAT+ and aSYN+ cells relative to TH-labeled cells in POLG1 variant and healthy control cultures, shown per differentiation. (C) Proportion of MAP2+ and GFAP+ cells out of DAPI in control and POLGQ811R dissociated cultures. [file 40478_2019_863_MOESM2_ESM.jpg]

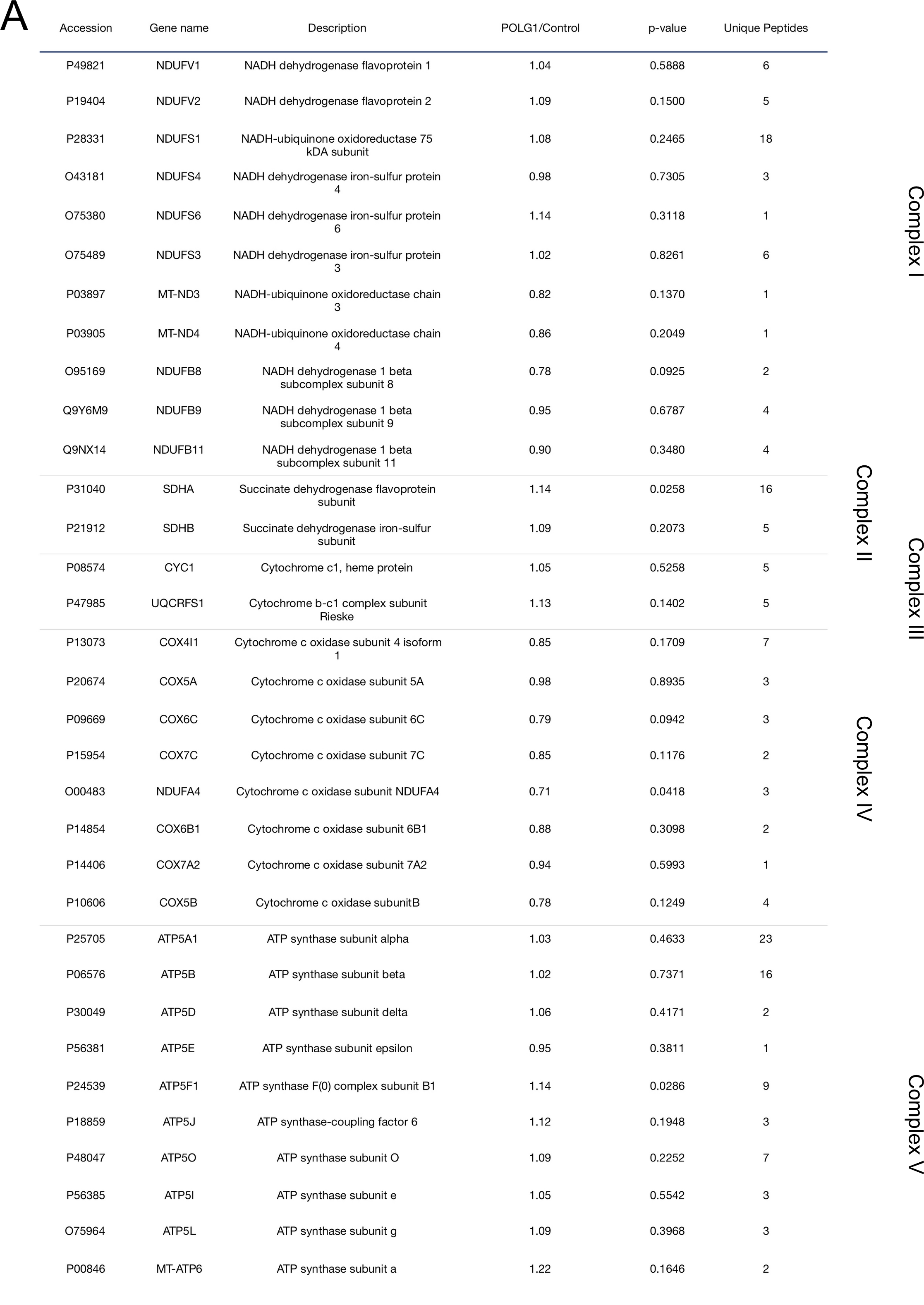

Supplement: Supplementary file 3 — Additional file 3: Figure S2. Abundance levels of proteins associated with subunits of complex I-IV of mitochondrial respiratory chain. [file 40478_2019_863_MOESM3_ESM.jpg]
